# Feasibility of short imaging protocols for [^18^F]PI-2620 tau-PET in progressive supranuclear palsy

**DOI:** 10.1007/s00259-021-05391-3

**Published:** 2021-05-22

**Authors:** Mengmeng Song, Maximilian Scheifele, Henryk Barthel, Thilo van Eimeren, Leonie Beyer, Ken Marek, Florian Eckenweber, Carla Palleis, Lena Kaiser, Anika Finze, Maike Kern, Alexander Nitschmann, Gloria Biechele, Sabrina Katzdobler, Gèrard Bischof, Jochen Hammes, Frank Jessen, Dorothee Saur, Matthias L. Schroeter, Jost-Julian Rumpf, Michael Rullmann, Andreas Schildan, Marianne Patt, Bernd Neumaier, Andrew W. Stephens, Boris-Stephan Rauchmann, Robert Perneczky, Johannes Levin, Joseph Classen, Günter U. Höglinger, Peter Bartenstein, Guido Boening, Sibylle Ziegler, Victor Villemagne, Alexander Drzezga, John Seibyl, Osama Sabri, Matthias Brendel, for the German Imaging Initiative for Tauopathies (GII4T)

**Affiliations:** 1 Department of Nuclear Medicine, University Hospital of Munich, LMU Munich, Marchioninstraße 15, 81377 Munich, Germany; 2 Department of Nuclear Medicine, University Hospital Leipzig, Leipzig, Germany; 3 Cognitive Neuroscience, Institute for Neuroscience and Medicine (INM-3), Research Centre Juelich, Juelich, Germany; 4 Department of Nuclear Medicine, University Hospital Cologne, Cologne, Germany; 5 Department of Neurology, University Hospital Cologne, Cologne, Germany; 6 German Center for Neurodegenerative Diseases (DZNE), Bonn, Germany; 7 InviCRO, LLC, Boston, MA USA; 8 Molecular Neuroimaging, A Division of inviCRO, New Haven, CT USA; 9 Department of Neurology, University Hospital of Munich, LMU Munich, Munich, Germany; 10 Radiologische Allianz, Nuklearmedizin Spitalerhof, Hamburg, Germany; 11 Department of Psychiatry, University Hospital Cologne, Cologne, Germany; 12 Center for Memory Disorders, University Hospital Cologne, Cologne, Germany; 13 Department of Neurology, University Hospital Leipzig, Leipzig, Germany; 14 Clinic for Cognitive Neurology, University Hospital Leipzig, Leipzig, Germany; 15 LIFE - Leipzig Research Center for Civilization Diseases, University of Leipzig, Leipzig, Germany; 16 Max- Planck-Institute of Human Cognitive and Brain Sciences, Leipzig, Germany; 17 Institute of Neuroscience and Medicine, INM-5: Nuclear Chemistry, Forschungszentrum Jülich GmbH, Jülich, Germany; 18 Institute of Radiochemistry and Experimental Molecular Imaging, University Hospital of Cologne, Cologne, Germany; 19 Life Molecular Imaging GmbH, Berlin, Germany; 20 Department of Psychiatry and Psychotherapy, University Hospital, LMU Munich, Munich, Germany; 21 Department of Radiology, University Hospital of Munich, LMU, Munich, Germany; 22 Ageing Epidemiology Research Unit (AGE), School of Public Health, Imperial College, London, UK; 23 German Center for Neurodegenerative Diseases (DZNE), Munich, Germany; 24 Munich Cluster for Systems Neurology (SyNergy), Munich, Germany; 25 Department of Neurology, Medizinische Hochschule Hannover, Hannover, Germany; 26 Department of Molecular Imaging & Therapy, Austin Health, Heidelberg, VIC Australia; 27 Department of Psychiatry, University of Pittsburgh, Pittsburgh, PA USA; 28 Department of Medicine, Austin Health, The University of Melbourne, Melbourne, VIC Australia

**Keywords:** Tau-PET, [^18^F]PI-2620, Time window, Progressive supranuclear palsy

## Abstract

**Purpose:**

Dynamic 60-min positron emission tomography (PET) imaging with the novel tau radiotracer [^18^F]PI-2620 facilitated accurate discrimination between patients with progressive supranuclear palsy (PSP) and healthy controls (HCs). This study investigated if truncated acquisition and static time windows can be used for [^18^F]PI-2620 tau-PET imaging of PSP.

**Methods:**

Thirty-seven patients with PSP Richardson syndrome (PSP-RS) were evaluated together with ten HCs. [^18^F]PI-2620 PET was performed by a dynamic 60-min scan. Distribution volume ratios (DVRs) were calculated using full and truncated scan durations (0–60, 0–50, 0–40, 0–30, and 0–20 min p.i.). Standardized uptake value ratios (SUVrs) were obtained 20–40, 30–50, and 40–60 min p.i.. All DVR and SUVr data were compared with regard to their potential to discriminate patients with PSP-RS from HCs in predefined subcortical and cortical target regions (effect size, area under the curve (AUC), multi-region classifier).

**Results:**

0–50 and 0–40 DVR showed equivalent effect sizes as 0–60 DVR (averaged Cohen’s d: 1.22 and 1.16 vs. 1.26), whereas the performance dropped for 0–30 or 0–20 DVR. The 20–40 SUVr indicated the best performance of all static acquisition windows (averaged Cohen’s d: 0.99). The globus pallidus internus discriminated patients with PSP-RS and HCs at a similarly high level for 0–60 DVR (AUC: 0.96), 0–40 DVR (AUC: 0.96), and 20–40 SUVr (AUC: 0.94). The multi-region classifier sensitivity of these time windows was consistently 86%.

**Conclusion:**

Truncated and static imaging windows can be used for [^18^F]PI-2620 PET imaging of PSP. 0–40 min dynamic scanning offers the best balance between accuracy and economic scanning.

**Supplementary Information:**

The online version contains supplementary material available at 10.1007/s00259-021-05391-3.

## Introduction

Progressive supranuclear palsy (PSP) is a neurodegenerative movement disorder characterized by pathological aggregation of hyperphosphorylated microtubule-associated four repeat (4R) isoform tau-protein in neurons and glial cells of the brain [1].

Clinical diagnosis of PSP only shows limited sensitivity and moderate specificity in early disease stages as revealed by recent autopsy-controlled data [2]. Also, since the development of tau targeting therapies is progressing at a high pace, the identification of specific biomarkers that would allow for early detection of tau pathology in PSP becomes crucial. An ideal biomarker would ensure that tau targeting therapies could be initiated as early as possible which may prove to be critical for an effective treatment of neurodegenerative diseases [3]. While current tau targeting trials in PSP include patients in later disease stages, a validated PSP tau biomarker could allow the inclusion of early-stage patients without loss of specificity.

The novel second-generation tau-PET tracer [^18^F]PI-2620 demonstrated high-affinity binding to isolated 4R tau fibrils and to PSP brain homogenates [4] which highlights its potential for imaging of 4R-tauopathies when compared to most other next-generation tau-PET tracers that are mainly specific for AD-tau [5]. Furthermore, the tracer indicated only very limited off-target binding to monoamine oxidases [4]. In our previous study, [^18^F]PI-2620 showed promising results for autoradiography assessment of PSP tissue in vitro and imaging of patients with PSP in vivo [6]. Dynamic [^18^F]PI-2620 imaging over 1 h already proved a high sensitivity to detect patients with PSP at a high specificity towards healthy controls and tau-negative neurodegeneration disorders [6].

Hence, this biomarker could be interesting for screening and monitoring of specific drug trials in PSP. Tau targeting therapeutics in PSP under current investigation, such as the tau aggregation inhibitors anle138b [7–9] and NPT088 [10, 11] as well as anti-Tau monoclonal antibodies like Gosuranemab [12, 13] and UCB0107 [14, 15], showed promising results and would probably profit from a reliable tau biomarker in potential phase II and phase III studies.

Despite the excellent diagnostic performance of [^18^F]PI-2620 in PSP when using a full dynamic setting of a 1-h scan [6], such long-lasting protocols are challenging for patients and cost-intensive in such trials. Therefore, we aimed to investigate the suitability of shorter dynamic or static acquisition protocols for [^18^F]PI-2620 tau-PET imaging in clinically diagnosed patients with PSP Richardson syndrome (PSP-RS). Given the fast tracer kinetics of [^18^F]PI-2620 and an inverted U-shape of relative binding in PSP target regions [6], we hypothesized that shorter dynamic scans and early static imaging windows provide equivalent discrimination of patients with PSP against controls when compared to a dynamic 1-h scan.

## Material and methods

### Study design and patient selection

Thirty-seven subjects with probable or possible PSP-RS according to current diagnostic criteria [16] as well as ten age- and gender-matched healthy controls (HC) were included in the primary analysis of this study. All participants were recruited and scanned at five different specialized centers in three countries (Munich, PSP-RS *n* = 20; Leipzig, PSP-RS *n* = 11; Cologne, PSP-RS n = 2; New Haven, PSP-RS *n* = 4, HC *n* = 5; Melbourne, HC n = 5), and all 0–60 min dynamic data were reported previously [6]. The participants were either scanned in a clinical setting or participated in the first in human study of [^18^F]PI-2620 [17]. Three of the initial 40 datasets were excluded due to missing listmode data which did not allow reconstruction of correct static frames. All participants (or their legal representatives) provided a written consent for PET imaging. The study protocol and PET data analyses were approved by the local ethics committee (LMU Munich, application numbers 17-569 and 19-022). The study was carried out according to the principles of the Helsinki Declaration. Additionally, we included β-amyloid-positive patients with typical AD (*n* = 11; age: 68.5 ± 6.9 years; 8 female; MMSE: 18.9 ± 7.1), as well as patients with probable PD (*n* = 6; age: 60.0 ± 9.8; 2 female; UPDRS: 22.5 ± 6.3; MoCA: 26.7 ± 4.1) and MSA-C (*n* = 4; age: 62.8 ± 5.8; 1 female; UPDRS: 26.0 ± 6.3; MoCA: 23.3 ± 3.8), all scanned in Munich, to test if suitable time windows for imaging of patients with PSP are also applicable to AD and α-synucleinopathies.

### PET imaging

#### Radiosynthesis

Radiosynthesis of [^18^F]PI-2620 was achieved by nucleophilic substitution on a BOC-protected nitro precursor using an automated synthesis module (IBA Synthera, Louvain-la-neuve, Belgium). The protecting group was cleaved under the radiolabelling conditions. The product was purified by semipreparative HPLC. Radiochemical purity was ≥97%. Non-decay corrected yields were about 30% with a molar activity of about 3∙10^6^ GBq/mmol at the end of synthesis.

#### Acquisition, reconstruction, and image harmonization

[^18^F]PI-2620 PET imaging was performed with different scanners using each established standard parameter at five specialized neuroimaging sites as described previously [6]. In brief, subjects were administered a single dose of [^18^F]PI-2620 (range 168–334 MBq) through venous catheter, followed by a 10 ml saline flush. Immediately following the intravenous injection (~10 s), continuous brain imaging was performed in a full dynamic setting (0–60 min p.i.). The original dynamic PET data were reconstructed into a series of 23 frames (6 × 30 s, 4 × 60 s, 4 × 120 s, and 9 × 300 s) and binned into single static frames of 20-min duration ranging from 20 to 40 min, 30 to 50 min, and 40 to 60 min p.i. Scanner-specific filter functions, which were obtained from Hofmann phantoms, were used to generate images with a similar resolution (FWHM: 9 × 9 × 10 mm), following the ADNI image harmonization procedure [18]. All dynamic images were visually checked and, if necessary, automatically corrected for head motion or non-standard posture (excessive head hypokinesis) before processing.

#### Image processing

Template generation, spatial normalization, and image preprocessing were performed as described previously [6]. In brief, a [^18^F]PI-2620 template was generated with 20 randomly selected datasets from PSP patients, disease controls, and healthy controls. Using the non-linear brain normalization function, all dynamic and static datasets were transformed to the MNI space via the transformation matrix of a 30–60 min template normalization.

Each full dynamic dataset (0–60 min) was truncated into a series of shorter durations (0–50, 0–40, 0–30, and 0–20 min p.i.). The cerebellum, excluding the dentate nucleus, the central cerebellar white matter, and the superior and the posterior cerebellar layers (d = 1.5 cm each), served as the reference region for calculation of distribution volume ratios (DVR) and standardized uptake value ratios (SUVr).

### PET data analysis and visual inspection

#### Definition of volumes of interests (VOIs)

For the PSP analysis, a total of nine predefined cortical and subcortical VOIs (dorsolateral and medial prefrontal cortex, internal and external part of the globus pallidus, the putamen, the subthalamic nucleus, the substantia nigra, the dorsal midbrain, and the dentate nucleus) derived from the Hammers and ATAG atlases [19, 20] were delineated in the MNI space. For the AD analysis, seven target regions were selected according to Braak stage atlas [21] (superior temporal gyrus, STG; primary visual cortex, PVC; middle temporal gyrus, MTG; fusiform gyrus, FUS; extrastriate visual cortex, EVC; entorhinal cortex, ERC; anterior hippocampus, AHC) and regional mean DVR/SUVr values (DVR 0–60 min, 0–40 min, and SUVr 20–40 min) were compared against HC.

#### Extraction of quantitative parameters

The multilinear reference tissue model 2 (MRTM2) [22] was used to generate parametric DVR (DVR = BPND + 1) images of the full 0–60 min and each truncated dynamic dataset (0–50, 0–40, 0–30, and 0–20 min p.i.). In addition, SUVr were obtained from static images (20–40, 30–50, and 40–60 min p.i.). All image data were processed and analyzed with PMOD (Version 3.4, PMOD Technologies Ltd., Zurich, Switzerland).

### Statistics

All group comparisons between patients with PSP-RS and healthy controls were performed separately in the nine predefined target regions: (I) Regional [^18^F]PI-2620 DVR and SUVrs of all different dynamic and static datasets were compared between PSP-RS and healthy controls using an unpaired two-tailed Student’s t test with adjustment for age and sex. *P* values were false discovery rate (FDR) corrected for multiple comparisons in nine VOIs. (II) Effect sizes (Cohen’s d) were calculated for the comparison of PSP-RS patients and controls. Negative Cohen’s d values were multiplied by −1 for comparability purposes. (III) A receiver-operating characteristic (ROC) curve analysis was performed to obtain the discriminative power for the comparison of PSP-RS patients and healthy controls by the area under the ROC curve (AUC). (IV) The sensitivity for detection of PSP-RS was calculated by a previously established multi-region classifier [6]. In this semi-quantitative analysis, a regional DVR/SUVr ≥ mean value (MV) + 2 standard deviations (SD) of the healthy controls was defined as positive. Here, one positive target region defined the subject as positive (dichotomous) for a PSP-like [^18^F]PI-2620 PET scan.

AUC values of all target regions were compared between short acquisition windows and 0–60 DVR by a paired t-test.

Pearson’s correlation coefficient (R) was used to determine the agreement between all short acquisition windows and 0–60 DVR as the standard of truth. The correlation analysis was performed for all nine target regions of PSP-RS patients. The deviation from the line of identity (y = x) was computed by the root-mean-square-error (RMSE) of all single patient measures.

The statistical analysis of patients with AD, α-synucleinopathies, and healthy controls was performed equally, except using different target regions for patients with AD and restriction to the following time windows: 0–60 DVR, 0–40 DVR, and 20–40 SUVr.

The significance level of *p* < 0.05 was applied in all analyses. All statistical analyses were carried out with GraphPad Prism 8 (GraphPad Software, San Diego, USA).

## Results

### Demographics and visual assessment of DVR and SUVr images

A total of 37 patients (15 female) with suspected PSP-RS according to current diagnosis criteria were included in the analysis. Patients (70.8 ± 6.3 years) and healthy controls (67.0 ± 7.4 years, 8 female) did not differ for age (*p* = 0.109; t-test) and had a slight difference in sex (*p* = 0.027; Χ^2^-test).

**Fig. 1:**
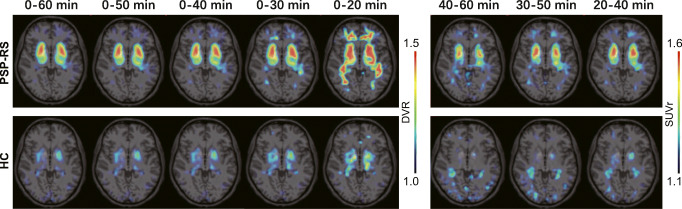
Representative [^18^F]PI-2620 images for different dynamic and static imaging windows. Axial slices upon an MRI standard template of a patient with PSP Richardson syndrome (PSP-RS; female, 69 years, PSP rating scale: 34) and a healthy control (HC; female, 70 years) show distribution volume ratios (DVR) and standardized uptake value ratios (SUVr)

The visual inspection of [^18^F]PI-2620 DVR images revealed strong artifacts for 0–20 DVR. Here, high binding in the subcortical white matter and implausibly high DVR values of single voxels (DVR > 10) were detected in many cases (see Fig. 1). Therefore, the 0–20 DVR window was excluded from further quantitative analysis. [^18^F]PI-2620 DVR and SUVr maps deriving from all other time windows provided valid patterns of tracer binding by qualitative visual assessment, but the distinguishable pattern in target regions appeared lower for 30–50 and 40–60 SUVr. Late phase SUVr images of patients with PSP-RS and HC showed higher relative white matter uptake when compared to DVR images. Representative [^18^F]PI-2620 DVR and SUVr images of all different time windows are shown for a patient with PSP-RS and a healthy control in Fig. 1.

### Quantitative comparison of truncated dynamic acquisitions against full dynamic acquisition

**Fig. 2:**
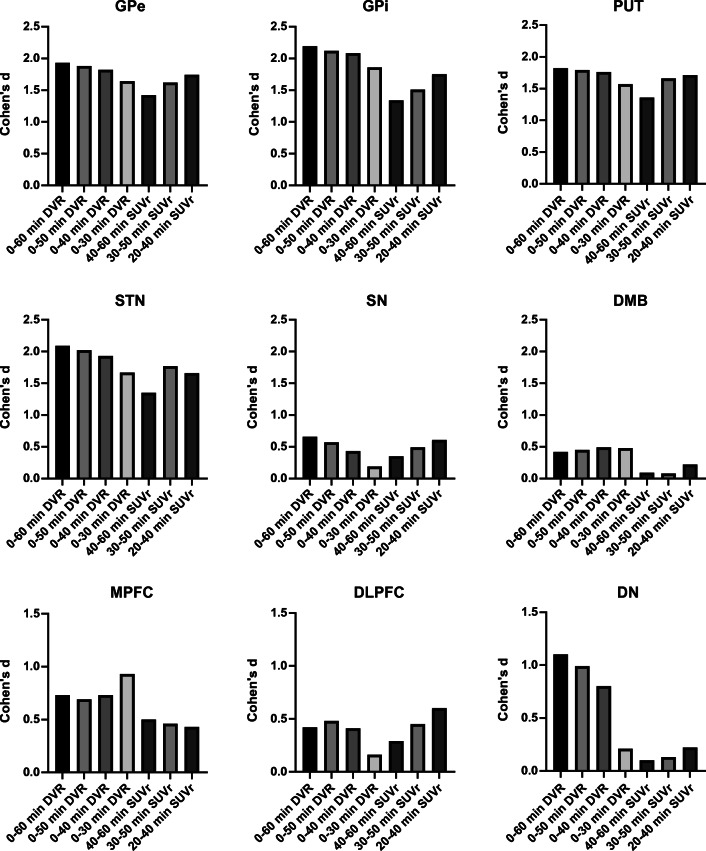
Effect sizes (Cohen’s d) in all brain regions for different dynamic and static [^18^F]PI-2620 imaging windows. DVR, distribution volume ratio; SUVr, standardized uptake value ratio; GPe, globus pallidus externus; GPi, globus pallidus internus; PUT, putamen; STN, subthalamic nucleus; SN, substantia nigra; DMB, dorsal midbrain; MPFC, medial prefrontal cortex; DLPFC, dorsolateral prefrontal cortex; DN, dentate nucleus. Negative Cohen’s d values were multiplied by −1 for comparability purposes

[^18^F]PI-2620 mean DVR and SUVr values of patients with PSP-RS and healthy controls of all acquisition windows are presented in Table 1 for nine target regions. Individual DVR and SUVr data points for all analyzed time windows in representative target regions are provided in the Supplement. Effect sizes (Cohen’s d) for all comparisons between patients with PSP and HC are visualized in Fig. 2. Different dynamic [^18^F]PI-2620 imaging windows showed nearly equal effect sizes for discriminating PSP and HC across all target regions for 0–60, 0–50, and 0–40 DVR but noticeably lower effect sizes for dynamic image acquisition times shorter than 40 min p.i. (0–30 DVR). A consistent magnitude of effect size was found for different dynamic windows (> 30 min p.i.) in the basal ganglia regions (GPi, GPe, PUT, STN), where the GPi performed continuously best (all Cohen’s d > 2.0). In the midbrain regions (SN and DMB) and the cortical regions (MPFC, DLPFC), we observed consistently lower effect sizes when compared to the basal ganglia but again at a similar level for all dynamic windows >30 min p.i.. In the dentate nucleus, longer scan duration comprised a larger effect size (0–60 DVR: Cohen’s d = 1.11) with a decrease towards shorter scan duration (0–40 DVR: Cohen’s d = 0.80). In summary, shortening the dynamic scan duration to 0–40 DVR provided nearly equivalent effect sizes for the contrast of PSP and HC when compared to a 1-h scan.

**Table 1:** DVR and SUVr mean values (± standard deviation) for PSP and HC for different dynamic and static [^18^F]PI-2620 imaging windows

		0–60 DVR	0–50 DVR	0–40 DVR	0–30 DVR	40–60 SUVr	30–50 SUVr	20–40 SUVr
GPe	PSP	1.15 ± 0.09	1.16 ± 0.09	1.16 ± 0.09	1.18 ± 0.10	1.21 ± 0.13	1.26 ± 0.12	1.29 ± 0.12
	HC	0.99 ± 0.05	1.00 ± 0.05	1.01 ± 0.05	1.03 ± 0.05	1.03 ± 0.09	1.08 ± 0.08	1.09 ± 0.07
	p value	<0.001	<0.001	<0.001	<0.001	<0.001	<0.001	<0.001
GPi	PSP	1.21 ± 0.09	1.22 ± 0.10	1.22 ± 0.10	1.24 ± 0.10	1.27 ± 0.16	1.34 ± 0.14	1.37 ± 0.13
	HC	1.00 ± 0.08	1.02 ± 0.07	1.04 ± 0.07	1.07 ± 0.07	1.07 ± 0.12	1.13 ± 0.10	1.15 ± 0.08
	p value	<0.001	<0.001	<0.001	<0.001	0.001	0.001	<0.001
PUT	PSP	1.16 ± 0.09	1.17 ± 0.09	1.17 ± 0.09	1.18 ± 0.10	1.14 ± 0.11	1.19 ± 0.11	1.23 ± 0.12
	HC	1.01 ± 0.06	1.02 ± 0.05	1.02 ± 0.05	1.04 ± 0.04	0.99 ± 0.10	1.02 ± 0.08	1.04 ± 0.08
	p value	<0.001	<0.001	<0.001	<0.001	0.001	<0.001	<0.001
STN	PSP	1.20 ± 0.08	1.21 ± 0.08	1.21 ± 0.08	1.23 ± 0.09	1.20 ± 0.12	1.25 ± 0.10	1.28 ± 0.10
	HC	1.03 ± 0.09	1.04 ± 0.08	1.05 ± 0.09	1.07 ± 0.10	1.04 ± 0.12	1.08 ± 0.09	1.12 ± 0.08
	p value	<0.001	<0.001	<0.001	<0.001	0.001	<0.001	<0.001
SN	PSP	1.16 ± 0.10	1.16 ± 0.09	1.16 ± 0.09	1.16 ± 0.10	1.38 ± 0.16	1.38 ± 0.15	1.34 ± 0.14
	HC	1.10 ± 0.08	1.11 ± 0.08	1.12 ± 0.08	1.18 ± 0.14	1.33 ± 0.13	1.31 ± 0.11	1.26 ± 0.08
	p value	0.106	0.158	0.298	0.637	0.601	0.366	0.219
DMB	PSP	0.87 ± 0.12	0.86 ± 0.12	0.85 ± 0.12	0.85 ± 0.12	1.04 ± 0.13	1.00 ± 0.13	0.94 ± 0.12
	HC	0.92 ± 0.10	0.91 ± 0.10	0.91 ± 0.10	0.91 ± 0.10	1.03 ± 0.14	1.01 ± 0.12	0.97 ± 0.11
	p value	0.120	0.106	0.088	0.109	0.626	0.293	0.202
MPFC	PSP	0.85 ± 0.08	0.85 ± 0.08	0.84 ± 0.08	0.84 ± 0.08	0.95 ± 0.12	0.94 ± 0.12	0.90 ± 0.10
	HC	0.91 ± 0.08	0.90 ± 0.07	0.90 ± 0.08	0.92 ± 0.11	1.01 ± 0.07	0.99 ± 0.07	0.94 ± 0.08
	p value	0.068	0.082	0.074	0.032	0.147	0.197	0.276
DLPFC	PSP	0.94 ± 0.07	0.94 ± 0.07	0.94 ± 0.07	0.94 ± 0.07	1.02 ± 0.12	1.03 ± 0.11	1.01 ± 0.10
	HC	0.91 ± 0.05	0.91 ± 0.05	0.91 ± 0.05	0.92 ± 0.06	0.99 ± 0.05	0.99 ± 0.06	0.96 ± 0.07
	p value	0.231	0.200	0.254	0.589	0.434	0.286	0.130
DN	PSP	1.15 ± 0.06	1.15 ± 0.06	1.15 ± 0.07	1.16 ± 0.07	1.16 ± 0.09	1.19 ± 0.09	1.21 ± 0.08
	HC	1.08 ± 0.03	1.09 ± 0.03	1.10 ± 0.05	1.14 ± 0.09	1.15 ± 0.05	1.18 ± 0.03	1.19 ± 0.04
	p value	0.008	0.017	0.043	0.510	0.686	0.543	0.384

P values derive from an unpaired Student’s t test including false discovery rate correction for nine target regions and seven methods (*n* = 63 comparisons) as well as adjustment for age and sex. *DVR* distribution volume ratio, *SUVr* standardized uptake value ratio, *PSP* progressive supranuclear palsy, *HC* healthy control, *GPe* globus pallidus externus, *GPi* globus pallidus internus, *PUT* putamen, *STN* subthalamic nucleus, *SN* substantia nigra, *DMB* dorsal midbrain, *MPFC* medial prefrontal cortex, *DLPFC* dorsolateral prefrontal cortex, *DN* dentate nucleus

### Quantitative comparison of short static windows

Overall, [^18^F]PI-2620 SUVr acquired from 20 to 40 min p.i. revealed consistently higher effect sizes (Cohen’s d_MEAN_: 0.99) when compared to 30–50 min p.i. (Cohen’s d_MEAN_: 0.91, *p* = 0.041, paired t-test of nine target regions) or 40–60 min p.i. (Cohen’s d_MEAN_: 0.76, *p* = 0.0015, paired t-test of nine target regions). For basal ganglia regions, static imaging windows showed large effect sizes (Cohen’s d ≥ 1.34) with 20–40 SUVr performing close to dynamic imaging windows (i.e., GPe: 20–40 SUVr Cohen’s d = 1.74 vs. 0–60 DVR Cohen’s d = 1.92). Effect sizes dropped from early to late static imaging windows in a linear manner for most basal ganglia regions. In the midbrain, 20–40 and 30–50 SUVr of the SN was performed at a similar level of effect size when compared to dynamic imaging, whereas there was a worse performance of short late imaging windows for the DMB when compared to dynamic imaging. In cortical regions, there was a consistently lower effect size of short late imaging windows for the MPFC but a reasonable performance of 20–40 SUVr in the DLPFC when compared to dynamic imaging. All SUVr windows indicated a low effect size for quantification of the DN.

### Discriminatory power of dynamic and static acquisition windows

**Fig. 3:**
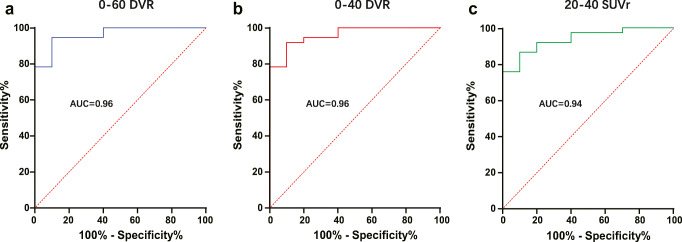
Receiver-operating characteristic (ROC) curve analysis in the globus pallidus internus. ROC curves show the discrimination of patients with progressive supranuclear palsy Richardson syndrome and healthy controls by globus pallidus internus quantification in different dynamic and static [^18^F]PI-2620 imaging windows. DVR, distribution volume ratio; SUVr, standardized uptake value ratio

Next, we performed a ROC analysis to evaluate the discrimination of patients with PSP from HC by regional [^18^F]PI-2620 quantification deriving from different time windows. Across all target regions, 0–50 DVR (mean AUC: 0.80, *p* = 0.336), 0–40 DVR (mean AUC: 0.79, *p* = 0.195), and 20–40 SUVr (mean AUC: 0.76, *p* = 0.136) showed no drop of the discriminatory power when compared to 0–60 DVR (mean AUC: 0.80) (Table 2). The ROC analysis of the basal ganglia target regions revealed the highest discriminatory power for all dynamic and static acquisition windows (AUC ≥ 0.82). Here, 0–40 DVR (AUC: 0.96/0.94) and 20–40 SUVr (AUC: 0.94/0.94) showed a similar discriminatory power for the internal and external part of the globus pallidus when compared to 0–60 DVR (AUC: 0.96/0.95). ROC curves of these time windows are illustrated in Fig. 3 for the internal part of the globus pallidus, and direct comparisons of all ROC curves are provided in the Supplement. Areas of the midbrain and the frontal cortex did not indicate AUC values sufficient to discriminate patients with PSP from HC regardless of the used imaging window (all AUC ≤ 0.72). The discriminatory power of the dentate nucleus was reasonable for 0–60 DVR (AUC: 0.80) but dropped for shorter dynamic scanning (AUC of 0–40 DVR: 0.73) or late static windows (AUC of 20–40 SUVr: 0.57).

**Table 2:** Comparison of area under the receiver-operating characteristic curve (AUC) values for the discrimination of patients with progressive supranuclear palsy from healthy controls

	0–60 DVR	0–50 DVR	0–40 DVR	0–30 DVR	40–60 SUVr	30–50 SUVr	20–40 SUVr
GPe	0.951	0.943	0.941	0.919	0.895	0.924	0.941
GPi	0.962	0.962	0.959	0.927	0.881	0.900	0.938
PUT	0.919	0.919	0.916	0.895	0.846	0.914	0.908
STN	0.930	0.924	0.916	0.868	0.824	0.911	0.905
SN	0.673	0.643	0.605	0.500	0.589	0.624	0.703
DMB	0.619	0.635	0.657	0.651	0.562	0.549	0.546
MPFC	0.722	0.719	0.714	0.714	0.700	0.686	0.633
DLPFC	0.661	0.669	0.656	0.550	0.600	0.644	0.694
DN	0.803	0.784	0.732	0.614	0.543	0.532	0.576
Mean AUC	0.804	0.800	0.788	0.738	0.716	0.743	0.760
*p*-value vs. 0–60 DVR		0.336	0.195	0.029	0.004	0.052	0.136

AUC values were calculated for all target regions and for all dynamic and static acquisition windows. *GPe* globus pallidus externus, *GPi* globus pallidus internus, *PUT* putamen, *STN* subthalamic nucleus, *SN* substantia nigra, *DMB* dorsal midbrain, *MPFC* medial prefrontal cortex, *DLPFC* dorsolateral prefrontal cortex, *DN* dentate nucleus, *DVR* distribution volume ratio, *SUVr* standardized uptake value ratio

### Performance of a multi-region classifier using dynamic and static acquisition windows

**Fig. 4:**
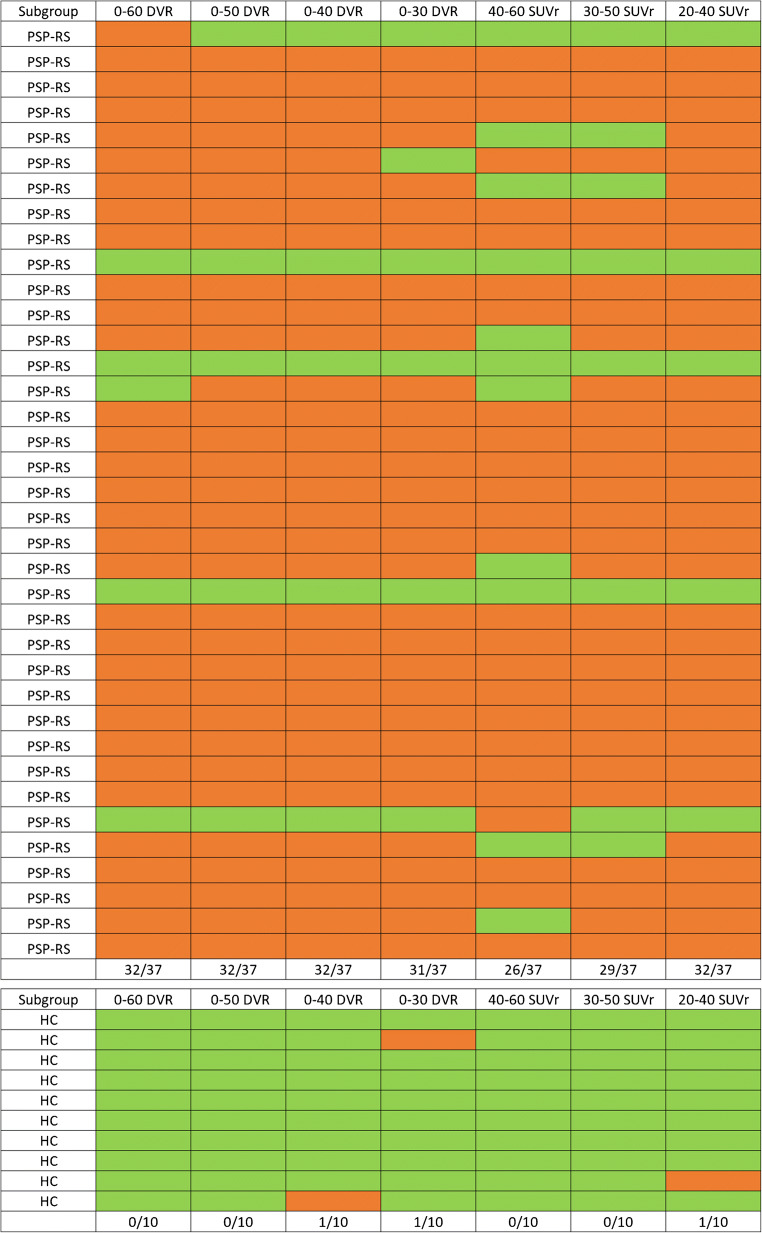
Multi-region classifier in comparison of dynamic and static imaging windows. Semi-quantitative classification (red, positive; green, negative) of PSP target regions was performed by applying a mean value (MV) + 2 standard deviations (SD) threshold as obtained from the healthy control (HC) data. One single region defined the scan as global positive, and only the global read-out is shown. Bottom rows provide the number of positive classified scans relative to the analyzed scans. PSP, progressive supranuclear palsy; RS, Richardson syndrome; DVR, distribution volume ratio; SUVr, standardized uptake value ratio

0–50 DVR, 0–40 DVR, and 20–40 SUVr showed an equal sensitivity of 86% when compared to 0–60 DVR. 0–30 DVR still showed a reasonable sensitivity of 83%, while static imaging at later time windows showed a noticeable loss in sensitivity (30–50 SUVr: 78%, 40–60 SUVr: 70%; see Fig. 4). Specificity was 90% or 100% in HC, indicating a maximum of one outlier HC regardless of the time window used.

### Quantitative agreement of short dynamic and static acquisition windows with 1-h dynamic scanning as a reference

The correlation coefficients determined by comparing the regional [^18^F]PI-2620 DVR and SUVr against 0–60 DVR and the resulting RMSE are shown in Table 3 and illustrated in the Supplement. The agreement of all dynamic imaging windows was excellent (R ≥ 0.906), whereas the agreement dropped for 30–50 SUVr (R ≥ 0.742) and 40–60 SUVr (R ≥ 0.614) when compared to 20–40 SUVr (R ≥ 0.865). This was also reflected by RMSE which revealed an overestimation of short window SUVr in contrast to 0–60 DVR (Table 3 & Supplement). Here, 20–40 SUVr indicated the slightest overestimation among the static short acquisition windows (RMSE 10.0% ± 3.6%), whereas there was a nearly perfect agreement of all truncated dynamic imaging windows (i.e., RMSE 0–40 DVR: 1.4% ± 0.4%).

**Table 3:** Correlation coefficients (R) and root-mean-square-errors (RMSE) in all brain regions for different dynamic and static [^18^F]PI-2620 imaging windows against 0–60 DVR as the reference

	0–50 DVR (R/RMSE)	0–40 DVR (R/RMSE)	0–30 DVR (R/RMSE)	40–60 SUVr (R/RMSE)	30–50 SUVr (R/RMSE)	20–40 SUVr (R/RMSE)
GPe	0.999/0.6%	0.994/1.3%	0.971/2.8%	0.680/9.7%	0.742/11.8%	0.868/13.1%
GPi	0.998/0.7%	0.992/1.6%	0.946/3.6%	0.730/10.4%	0.809/12.7%	0.893/14.2%
PUT	0.999/0.5%	0.995/1.2%	0.985/2.1%	0.699/7.3%	0.760/6.5%	0.899/7.4%
STN	0.998/0.6%	0.988/1.4%	0.933/3.4%	0.626/7.6%	0.774/6.5%	0.865/8.0%
SN	0.998/0.7%	0.989/1.4%	0.906/3.6%	0.787/20.7%	0.858/19.6%	0.911/16.3%
DMB	0.999/1.1%	0.997/2.2%	0.981/3.6%	0.849/21.3%	0.919/15.7%	0.976/8.9%
MPFC	0.999/0.6%	0.995/1.3%	0.962/2.8%	0.841/14.3%	0.898/11.9%	0.959/6.9%
DLPFC	0.996/0.7%	0.990/1.1%	0.948/2.5%	0.759/12.1%	0.847/12.0%	0.933/9.1%
DN	0.998/0.4%	0.992/0.9%	0.968/1.9%	0.614/6.3%	0.752/6.4%	0.884/6.5%

*GPe* globus pallidus externus, *GPi* globus pallidus internus, *PUT* putamen, *STN* subthalamic nucleus, *SN* substantia nigra, *DMB* dorsal midbrain, *MPFC* medial prefrontal cortex, *DLPFC* dorsolateral prefrontal cortex, *DN* dentate nucleus, *DVR* distribution volume ratio, *SUVr* standardized uptake value ratio

### Application of truncated dynamic imaging and short acquisition windows to [^18^F]PI-2620 imaging in AD and α-synucleinopathies

**Fig. 5:**
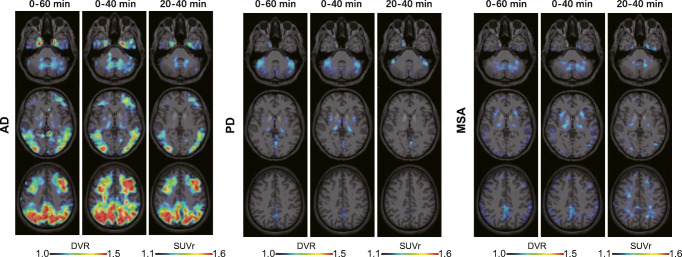
Representative [^18^F]PI-2620 images for 0–60 DVR, 0–40 DVR, and 20–40 SUVr in Alzheimer’s disease (AD) and α-synucleinopathies. Axial slices upon an MRI standard template show distribution volume ratios (DVR) and the standardized uptake value ratio (SUVr) of a patient with AD (female, 66 years, MMSE: 20), a patient with PD (female, 57 years, MoCA: 28, UPDRS: 25), and a patient with MSA-C (male, 55 years, MoCA: 26, UPDRS: 28)

Qualitative visual assessment provided similar tracer binding patterns for 0–40 DVR and when compared to 0–60 DVR as a reference (Fig. 5). For AD, 20–40 SUVr indicated similar patterns when compared to dynamic imaging but revealed slightly lower discernible binding in some AD target regions like the mesial temporal lobe (Fig. 5).

According to the PSP analyses above, effect sizes and AUC values were calculated for the comparison of AD and HC by use of AD target regions as well as for the comparison of PSP and α-synucleinopathies by use of the PSP target regions.

Regarding the effect sizes in AD (Table 4A), all target regions revealed very similar values for full and short acquisition windows except only a moderate agreement for the STG. The AUC values of the ROC analysis (Table 4B) revealed a high discriminatory power for [^18^F]PI-2620 between AD and HC for the PVC, MTG, FUS, EVC, and ERC in all acquisition windows, with the PVC and the ERC performing best. The quantitative agreement (Table 4C) of 0–40 DVR and 20–40 SUVr with 0–60 DVR was excellent (R ≥ 0.900) for all target regions except for ERC and AHC, where the agreement dropped slightly for the static acquisition window (ERC 20–40 SUVr: R = 0.886; AHC 20–40 SUVr: R = 0.771). The RMSEs revealed an overestimation for all imaging windows in contrast to 0–60 DVR. Dynamic imaging indicated a very good agreement (RMSE of 0–40 DVR: 3.2% ± 1.5%), while the static acquisition window showed a slight overestimation (RMSE of 20–40 SUVr: 10.7% ± 2.6%).

**Table 4:** Effect sizes (Cohen’s d), AUC values, and quantitative agreement with 0–60 DVR for all AD target regions for 0–40 DVR and 20–40 SUVr

A) Cohen’s d	STG	PVC	MTG	FUS	EVC	ERC	AHC
0–60	0.494	1.265	1.258	1.600	0.983	1.662	0.769
0–40	0.420	1.227	1.129	1.455	1.007	1.362	0.632
20–40	0.885	1.219	1.430	1.662	0.844	1.732	0.644
B) AUC	STG	PVC	MTG	FUS	EVC	ERC	AHC
0–60	0.682	0.900	0.800	0.859	0.889	0.900	0.727
0–40	0.627	0.836	0.791	0.899	0.889	0.863	0.682
20–40	0.764	0.936	0.845	0.899	0.798	0.909	0.682
C) R/RMSE	STG	PVC	MTG	FUS	EVC	ERC	AHC
0–40 DVR	0.948/3.5%	0.937/6.2%	0.976/2.8%	0.986/2.0%	0.975/2.7%	0.980/3.4%	0.941/1.8%
20–40 SUVr	0.900/7.3%	0.991/9.1%	0.960/8.1%	0.937/14.3%	0.926/11.0%	0.886/13.0%	0.771/11.9%

*STG* superior temporal gyrus, *PVC* primary visual cortex, *MTG* middle temporal gyrus, *FUS* fusiform gyrus, *EVC* extrastriate visual cortex, *ERC* entorhinal cortex, *AHC* anterior hippocampus, *DVR* distribution volume ratio, *SUVr* standardized uptake value ratio, *R* Pearson’s correlation coefficient, *RMSE* root-mean-square-error, *AUC* area under the curve

Discrimination of PSP and alpha-synucleinopathies was achieved at similar effect sizes for the full and short [^18^F]PI-2620 acquisition windows (Table 5A). Consistently with 0–60 DVR, the effect sizes of 0–40 DVR and 20–40 SUVr were highest in the basal ganglia regions (GPi, GPe, PUT, and STN), with the GPi performing the best (Cohen’s d ≥ 1.727). The ROC analysis (Table 5B) indicated a high discriminatory power for [^18^F]PI-2620 between PSP and alpha-synucleinopathies in the basal ganglia target regions (GPi, GPe, PUT, and STN), SN and DN for all investigated time windows, with the GPi performing best (AUC 0–60/0–40/20–40 = 0.98/0.97/0.95). The quantitative agreement (Table 5C) of 0–40 DVR with 0–60 DVR was excellent for all target regions (R ≥ 0.969), while the agreement decreased slightly for 20–40 SUVr (R = 0.848 ± 0.117). RMSEs revealed a nearly perfect agreement for 0–40 DVR (1.7% ± 0.7%) and a slight overestimation for 20–40 SUVr (8.3% ± 3.6%) when compared to 0–60 DVR.

**Table 5:** Effect sizes (Cohen’s d), AUC values, and quantitative agreement with 0–60 DVR for 0–40 DVR and 20–40 SUVr with the aim to discriminate PSP from α-synucleinopathies

A) Cohen’s d	GPe	GPi	PUT	STN	SN	DMB	MPFC	DLPFC	DN
0–60	1.579	1.961	1.317	1.428	0.927	0.293	0.784	0.209	1.120
0–40	1.487	1.986	1.228	1.381	0.891	0.258	0.782	0.276	1.114
20–40	1.348	1.727	1.284	1.531	1.143	0.131	0.410	0.436	1.128
B) AUC	GPe	GPi	PUT	STN	SN	DMB	MPFC	DLPFC	DN
0–60	0.903	0.976	0.822	0.870	0.765	0.643	0.744	0.561	0.805
0–40	0.892	0.973	0.797	0.876	0.749	0.624	0.742	0.581	0.819
20–40	0.859	0.949	0.843	0.878	0.843	0.589	0.661	0.619	0.814
C) R/RMSE	GPe	GPi	PUT	STN	SN	DMB	MPFC	DLPFC	DN
0–40 DVR	0.976/2.2%	0.978/2.4%	0.994/1.7%	0.978/1.9%	0.983/1.0%	0.991/2.5%	0.994/1.2%	0.969/1.0%	0.991/0.7%
20–40 SUVr	0.893/11.9%	0.754/12.6%	0.898/4.7%	0.621/7.1%	0.736/11.7%	0.956/6.6%	0.958/3.9%	0.889/11.3%	0.925/4.6%

*GPe* globus pallidus externus, *GPi* globus pallidus internus, *PUT* putamen, *STN* subthalamic nucleus, *SN* substantia nigra, *DMB* dorsal midbrain, *MPFC* medial prefrontal cortex, *DLPFC* dorsolateral prefrontal cortex, *DN* dentate nucleus, *DVR* distribution volume ratio, *SUVr* standardized uptake value ratio, *R* Pearson’s correlation coefficient, *RMSE* root-mean-square-error, *AUC* area under the curve

In summary, we observed a high agreement between 0 – 40 DVR and 0–60 DVR for [^18^F]PI-2620 imaging in patients with AD, whereas the performance of 20–40 SUVr slightly dropped in mesial temporal target regions. Differentiation of PSP and α-synucleinopathies was performed at an equal level for all investigated time windows.

## Discussion

In this study, we evaluated optimized acquisition times for [^18^F]PI-2620 tau-PET imaging in PSP. Both dynamic image acquisition over 40 min and static acquisition from 20 to 40 min post injection indicated an excellent performance when compared to full dynamic scanning over 1 h. We find that 0–40 DVR provide equivalent discrimination and quantification of [^18^F]PI-2620 PET in PSP when compared to 0–60 DVR, whereas 20–40 SUVr can be used for discrimination of patients with PSP with a moderate deviation of quantification. Furthermore, truncated dynamic scanning also showed feasibility in AD and for discrimination of PSP from α-synucleinopathies.

Recommendations derived from our data depend on the specific setting and the purpose of [^18^F]PI-2620 tau-PET imaging in PSP, which can be roughly divided in (I) therapy monitoring of tau-targeting therapies, (II) PET imaging as an inclusion criteria of clinical trial, (III) observational studies, and (IV) clinical differential diagnosis. We note that this is a preliminary opinion since large-scaled longitudinal studies with [^18^F]PI-2620 in PSP are not yet completed.

Therapy monitoring of anti-tau treatments in PSP will require a precise biomarker read-out that should allow to detect even subtle changes of the therapy target in vivo [23]. Furthermore, longitudinal studies will require a read-out that is only slightly affected by changes in cerebral blood flow [24]. Thus, dynamic imaging will be superior over short static windows for the purpose of longitudinal treatment monitoring. Our data indicate that 0–40 DVR provide highly congruent data when compared to 0–60 DVR; thus, a reduction of one-third of the scan duration is feasible without relevant loss of performance. Another advantage of dynamic [^18^F]PI-2620 acquisition is the possibility to acquire early phase or R1 images as a surrogate for neuronal injury [25].

One strength of PET is its ability to prove target presence before treatment initiation. This was impressively shown for β-amyloid PET which revealed post hoc that β-amyloid-modifying trials were initiated with more than one-third of β-amyloid-negative patients that could likely not profit from the therapy [26]. Consequently a positive β-amyloid PET was implemented as a screening criterion in many phase III trials, including the β-amyloid antibody aducanumab [27] and the beta-secretase inhibitors verubecestat and lanabecestat [28]. [^18^F]PI-2620 yielded a high sensitivity for detection of patients with PSP in our recent multi-center evaluation and could potentially serve as a screening criterion in anti-tau PSP trials [6]. In this regard, the discrimination of patients with PSP-RS from HC by [^18^F]PI-2620 was achieved at a similar level by DVR obtained from a dynamic 40 min scan and also by a short 20–40 min SUVr quantification when compared to 0–60 DVR. Furthermore, the sensitivity for detection of PSP-RS was consistently 86% when using a multi-region classifier with these different time windows. Since most trials will concomitantly use the screening scans as baseline, we primarily recommend 0–40 DVR for the purpose of patient screening. This time window also showed excellent performance for discrimination of PSP from α-synucleinopathies which is probably most relevant in the screening phase of trials. However, for the pure purpose of screening, 20–40 SUVr could serve for sufficient discriminatory power when dynamic scanning is not consistently available in large multi-center trials.

Observational studies (single or multiple time points) of PSP will likely follow the same requirements as monitoring studies. However, multi-tracer studies may require a trade-off between accuracy and patient effort to ensure the participants’ compliance, thus making short acquisition windows necessary in terms of study feasibility. Overestimations as a function of binding were found for all short [^18^F]PI-2620 SUVr windows when compared to DVR. However, we observed a still high correlation between 20 – 40 SUVr quantification and 0–60 DVR for most [^18^F]PI-2620 target regions of PSP. Furthermore, the resulting error of 20–40 SUVr with 0–60 DVR as a reference was of modest size. Therefore, 20–40 SUVr may be considered for observational [^18^F]PI-2620 studies in PSP when compromises need to be made with regard to the global patient effort of the investigation.

[^18^F]PI-2620 has not been investigated in a clinical differential diagnosis scenario of PSP patients and similar diseases yet. However, preliminary data indicated a different binding magnitude and different binding patterns of [^18^F]PI-2620 when comparing PSP against α-synucleinopathies and AD [6]. Since short static windows of [^18^F]PI-2620 provide similar binding patterns of PSP and HC when compared to a 1-h dynamic acquisition, they should facilitate comparable performance in differentiation of PSP from other diseases. In this regard, we investigated small samples of patients with AD and α-synucleinopathies and found a good performance of 0–40 DVR and an acceptable performance of 20–40 SUVr. [^18^F]PI-2620 shows a fast washout from non-target regions and increasing SUVrs over time in AD target regions [29], revealing optimal pseudoequilibrium, test-retest variability, and correlation with full tracer kinetics for late imaging windows. This led to recommendation of imaging between 30 and 90 min p.i. for AD [17, 29]. Our findings show that dynamic scanning can be reduced to 40 min with additional gain of the perfusion phase as a neuronal injury surrogate [25]. When detailed quantification is not needed in a pure clinical setting, 20–40 SUVr could also facilitate robust identification of AD tau pathology. However, it needs to be considered that the situation might be different for early stages of AD (i.e., Braak I/II), when a faint signal needs to be distinguished in the entorhinal cortex and the hippocampus. Here, we observed the most relevant drop of performance for 20–40 SUVr when compared to 0–60 DVR which is in line with the observation of increasing [^18^F]PI-2620 SUVR over time in AD even beyond 60 min p.i. [30]. Thus, we recommend truncated dynamic imaging (0–40 DVR) when the mesial temporal lobe is subject of evaluation. In comparison to the proposed imaging windows for [^18^F]MK-6240 (70–90 min [31]), [^18^F]flortaucipir (80–100 min [32]), [^18^F]RO-948 (70–90 min [33]), or [^18^F]PM-PBB3 (90–110 min [34]), the possibility of early scanning may pose an advantage for [^18^F]PI-2620 in a clinical setting, since the patients have a low attending time in a nuclear medicine department. We note that the capability of binding in non-AD tauopathies differs between next-generation tracers as [^18^F]MK-6240 and [^18^F]RO-948 both show a high specificity for AD tau aggregates, while they do not seem to significantly bind to non-AD tau aggregates [33, 35]. In contrast previous studies demonstrated that [^18^F]PI-2620 and [^18^F]PM-PBB3 show binding in AD and non-AD tauopathies [6, 34]. However, it needs to be taken into consideration that early time windows or dynamic imaging has not been performed for most of the tracer mentioned above. Thus, it could be possible that binding of these ligands in PSP has not been documented due to missing data in early time windows. We conclude that a final statement on the capability of these tau-PET tracers to measure non-AD tau in vivo cannot be obtained currently. The mechanism that makes early short windows like 20–40 SUVr more suitable for imaging of 4R-tauopathies than late short windows like 40–60 SUVr is not completely understood yet. However, we recently found higher k2/k2a values in assumed tau-positive clusters of 4R-tauopathies when compared to assumed tau-positive clusters of the 3/4R-tauopathy AD [36]. This could indicate a faster clearance from the target in 4R-tauopathies, and it could potentially explain the inverted U-shape of the time-SUVr curves in PSP [6].

We found some differences regarding the suitability of short acquisition windows for [^18^F]PI-2620 between PSP target regions. Basal ganglia regions, which show the highest effect sizes and the best discrimination rates for PSP against HC [6], consistently showed a good performance when using short dynamic scanning or short static windows. However, the dentate nucleus indicated a loss of effect size and discriminatory power as a function of truncation of dynamic scan time. This suggests that there could be a mixture between target binding and perfusion effect in the dentate nucleus. Regarding the substantia nigra, the off-target binding of [^18^F]PI-2620 to neuromelanin needs to be considered as a potential confounder. One HC was classified as positive for PSP in the 20–40 time window because of an isolated regional positivity of the substantia nigra. This suggests that the off-target binding variability of [^18^F]PI-2620 in the substantia nigra could be increased in short imaging windows. Since, none of the PSP cases was classified as PSP based on an isolated positivity of the substantia nigra, the inclusion of this target region should be interrogated carefully.

Our main results are limited to thirty-seven PSP patients and ten control subjects and need to be interpreted with appropriate caution. Yet our data hold promising results for the value of shorter [^18^F]PI-2620 imaging windows which should be confirmed in larger cohorts.

## Conclusions

Our data support the use of static 20–40 min or dynamic 0–40 min time intervals for [^18^F]PI-2620 PET imaging of PSP. Truncated dynamic acquisition over 40 min after tracer injection may also be suitable for [^18^F]PI-2620 PET imaging of AD tau pathology.

## Supplementary information

ESM 1 (DOCX 1515 kb)

ESM 2 (DOCX 12 kb)
