# Supplementary material for: Feasibility of short imaging protocols for [18F]PI-2620 tau-PET in progressive supranuclear palsy
Source: Eur J Nucl Med Mol Imaging. 2021 May 22;48(12):3872–85. doi: 10.1007/s00259-021-05391-3 (PMC8484138; doi:10.1007/s00259-021-05391-3)
Supplement: Supplementary file 1 — (DOCX 1515 kb) [file 259_2021_5391_MOESM1_ESM.docx]

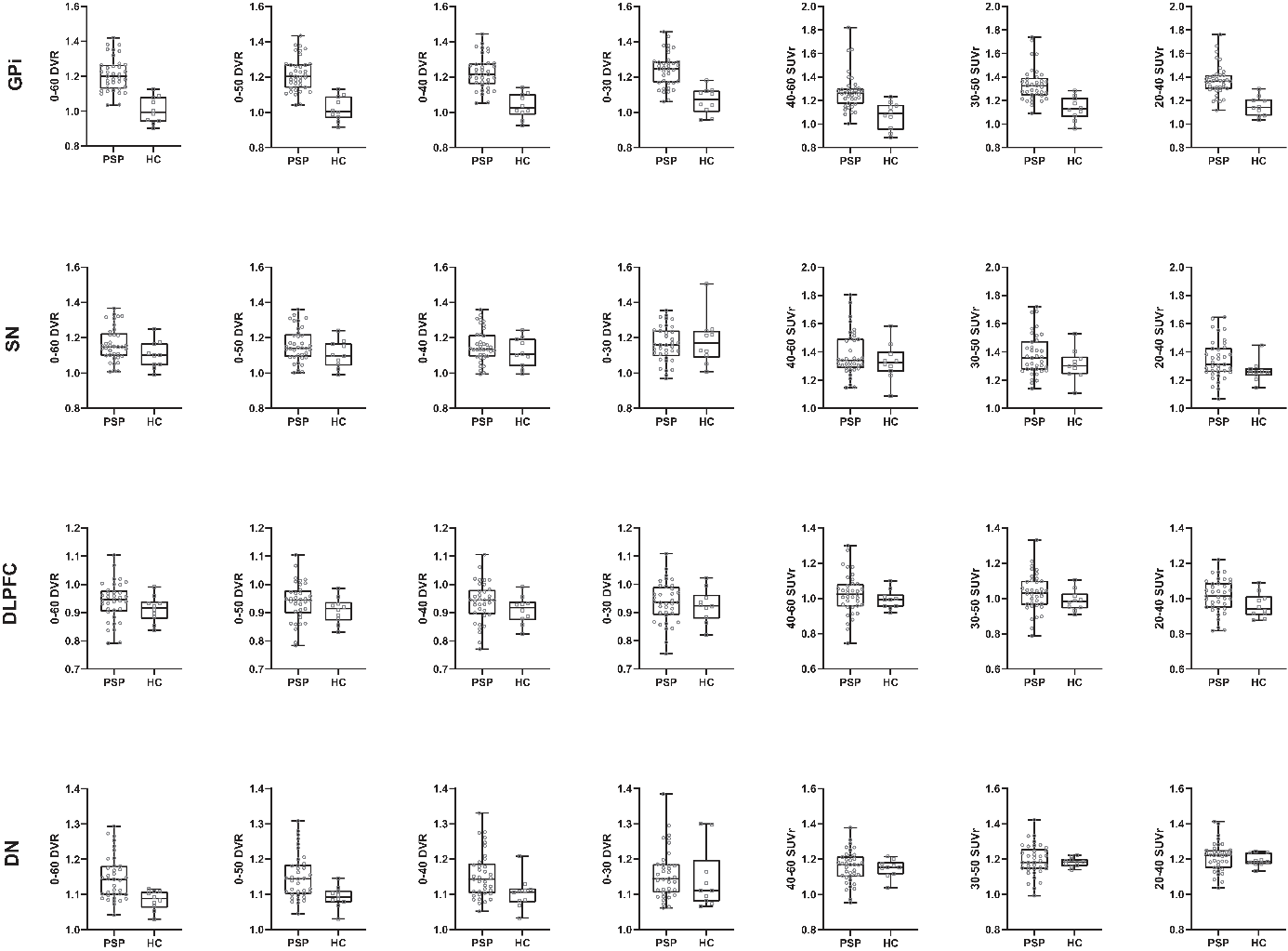


**Supplemental Figure 1: Illustration of box plots and individual DVR and SUVr data points of patients with progressive supranuclear palsy (PSP) and healthy controls (HC) in representative brain regions for different dynamic and static [^18^F]PI-2620 imaging windows.** DVR = distribution volume ratio; SUVr = standardized uptake value ratio; GPi = globus pallidus internus; SN = substantia nigra; DLPFC = dorsolateral prefrontal cortex; DN = dentate nucleus


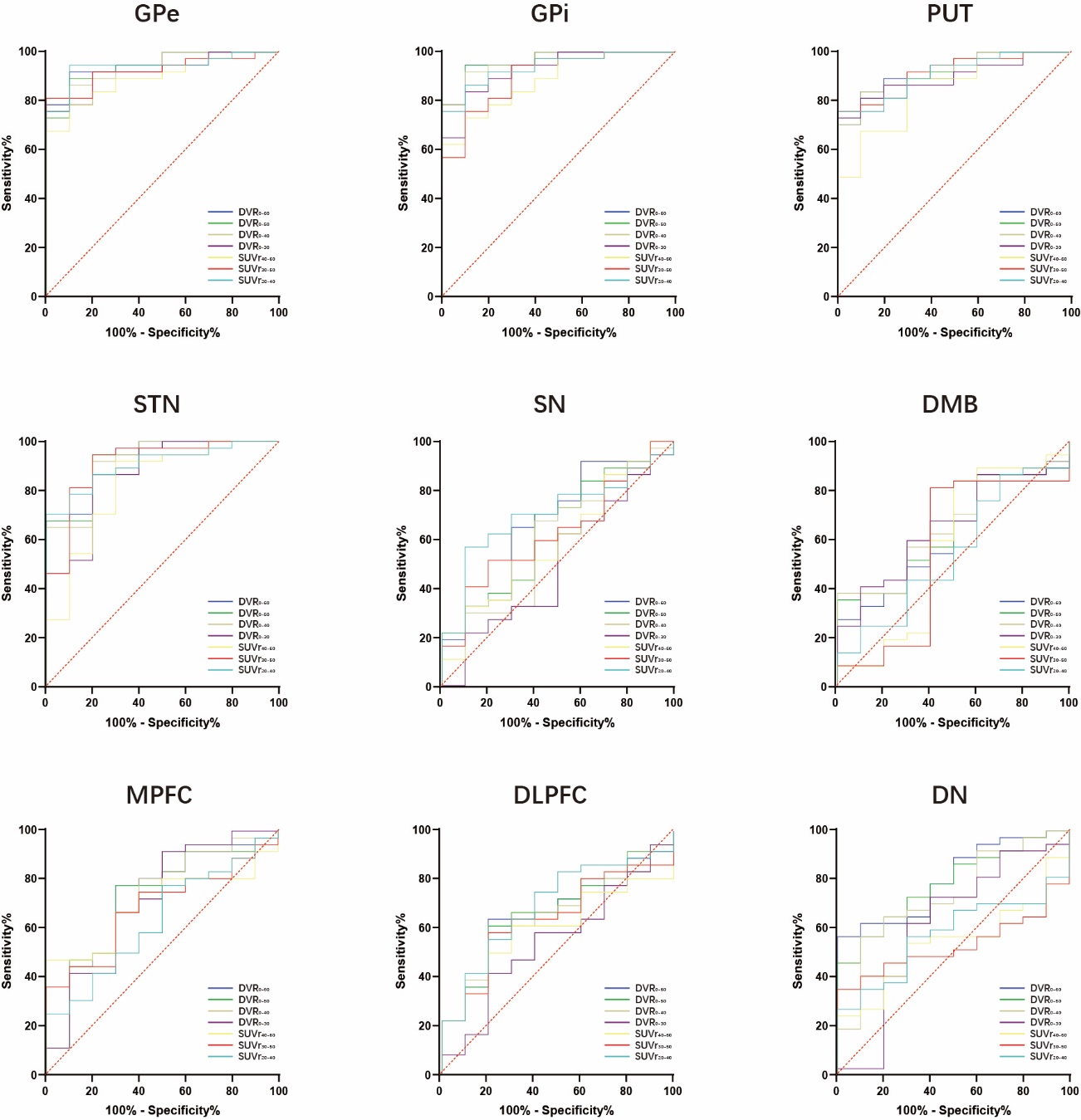


**Supplemental Figure 2: Illustration of receiver operating curves (ROC) in all brain regions for different dynamic and static [^18^F]PI-2620 imaging windows.** DVR = distribution volume ratio; SUVr = standardized uptake value ratio; GPe = globus pallidus externus; GPi = globus pallidus internus; PUT = putamen; STN = subthalamic nucleus; SN = substantia nigra; DMB = dorsal midbrain; MPFC = medial prefrontal cortex; DLPFC = dorsolateral prefrontal cortex; DN = dentate nucleus


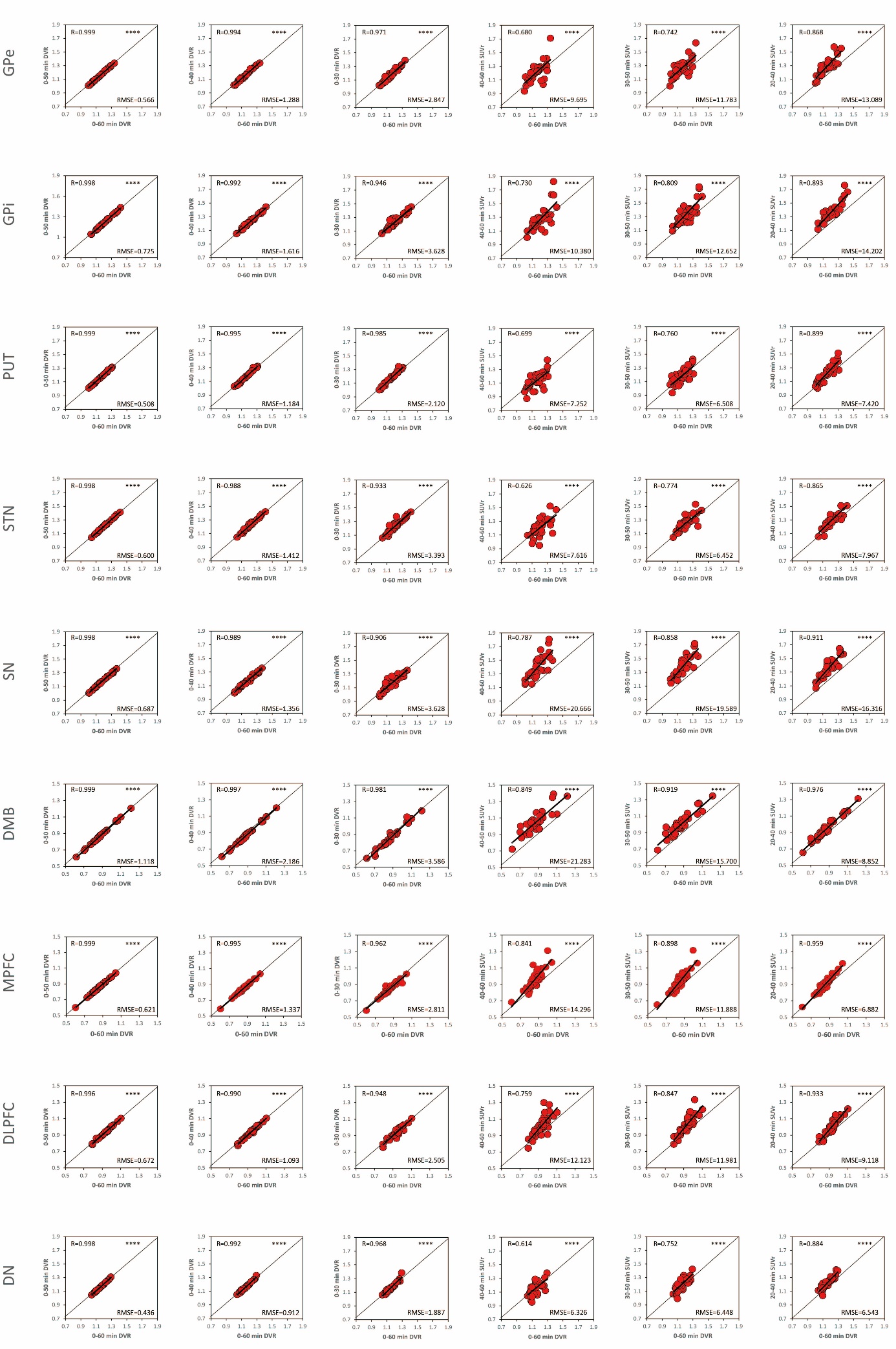


**Supplemental Figure 3: Agreement between different truncated dynamic and static [^18^F]PI-2620 imaging windows with 0-60 distribution volume ratios (DVR) for all brain regions.** SUVr = standardized uptake value ratio; GPe = globus pallidus externus; GPi = globus pallidus internus; PUT = putamen; STN = subthalamic nucleus; SN = substantia nigra; DMB = dorsal midbrain; MPFC = medial prefrontal cortex; DLPFC = dorsolateral prefrontal cortex; DN = dentate nucleus; RMSE = root-mean-square-error; R = Pearson’s correlation coefficient


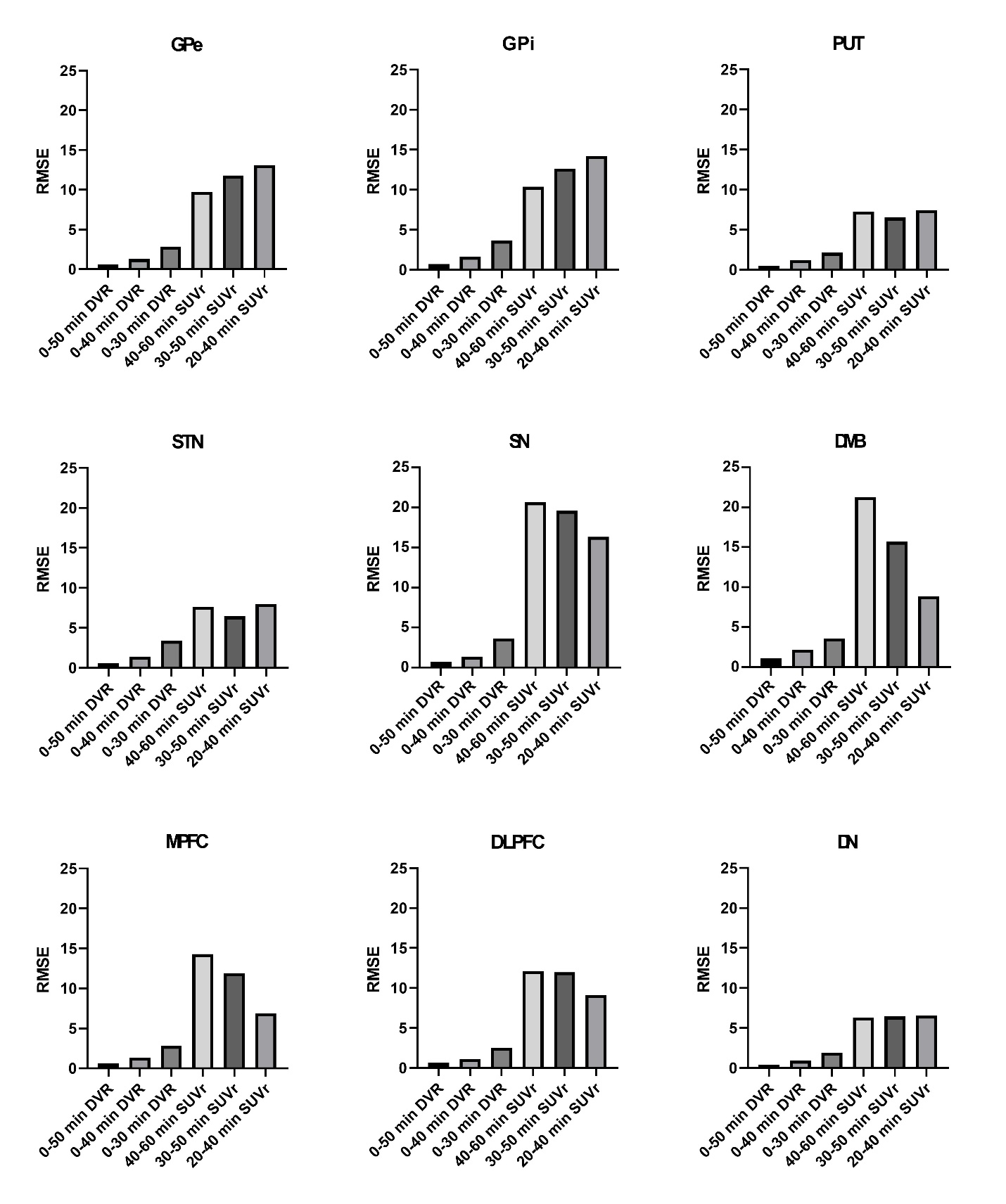


**Supplemental Figure 4: Illustration of root-mean-square-errors (RMSE) in all brain regions for different dynamic and static [^18^F]PI-2620 imaging windows.** DVR = distribution volume ratio; SUVr = standardized uptake value ratio; GPe = globus pallidus externus; GPi = globus pallidus internus; PUT = putamen; STN = subthalamic nucleus; SN = substantia nigra; DMB = dorsal midbrain; MPFC = medial prefrontal cortex; DLPFC = dorsolateral prefrontal cortex; DN = dentate nucleus; RMSE = root-mean-square-error
