# Supplementary material for: Feasibility of short imaging protocols for [18F]PI-2620 tau-PET in progressive supranuclear palsy
Source: Eur J Nucl Med Mol Imaging. 2021 May 22;48(12):3872–85. doi: 10.1007/s00259-021-05391-3 (PMC8484138; doi:10.1007/s00259-021-05391-3)
Supplement: Supplementary file 2 — (DOCX 12 kb) [file 259_2021_5391_MOESM2_ESM.docx]

**Appendix**

^#^**German Imaging Initiative for Tauopathies (GII4T)**

LMU Munich, Dept. Neurology: Johannes Levin, Jonathan Vöglein, Urban Fietzek, Sonja Schönecker, Georg Nübling, Catharina Prix, Kai Bötzel, Adrian Danek, Carla Palleis, Endy Weidinger, Sabrina Katzdobler

LMU Munich, Dept. Nuclear Medicine: Matthias Brendel, Mengmeng Song, Alexander Nitschmann, Maike Kern, Gloria Biechele, Anika Finze, Leonie Beyer, Peter Bartenstein, Stefanie Harris, Julia Schmitt, Florian Eckenweber, Simon Lindner, Franz-Joseph Gildehaus, Emanuel Joseph, Maximilian Scheifele, Christian Zach

LMU Munich, Dept. Psychiatry and Psychotherapy: Robert Perneczky, Jan Häckert

LMU Munich, Dept. Radiology: Boris-Stephan Rauchmann, Sophia Stöcklein

Hannover Medical School, Dept of Neurology: Günter Höglinger, Gesine Respondek

University of Leipzig, Dept. Nuclear Medicine: Henryk Barthel, Marianne Patt, Andreas Schildan, Osama Sabri, Michael Rullmann

University of Leipzig, Dept. of Neurology: Joseph Classen, Dorothee Saur, Jost-Julian Rumpf

Max-Plank-Institute of Human Cognitive and Brain Sciences Leipzig: Matthias L. Schroeter,

Technical University of Munich, Dept. Neurology: Matthias Höllerhage

University of Cologne, Dept. Nuclear Medicine and Forschungszentrum Jülich: Alexander Drzezga, Thilo van Eimeren, Jochen Hammes, Bernd Neumaier

University of Cologne, Dept. Neurology: Michael T. Barbe, Oezguer Onur

DZNE Munich/Bonn: Estrella Morenas-Rodriguez, Jochen Herms, Sigrun Roeber, Thomas Arzberger, Christian Haass, Frank Jessen

Life Molecular Imaging: Andrew Stephens, Norman Koglin, Andre Mueller
